# Incidence of symptomatic discoid meniscus in Korea: epidemiologic big data analysis from HIRA database

**DOI:** 10.1186/s43019-024-00234-5

**Published:** 2024-10-25

**Authors:** Jin Seong Kim, Jung Hoon Kim, Moon Young Choi, Jeong Ku Ha, Seung Hun Baek, Kyu Sung Chung

**Affiliations:** 1 Department of Physical Therapy, Ilsan Paik Hospital, College of Medicine, Inje University, Goyang-si, Gyeonggi-do, Republic of Korea; 2 Department of Physical Education, College of Humanities, Daegu University, Gyeongsan-si, Gyeongsangbuk-do, Republic of Korea; 3 Department of Orthopedic Surgery, Jump Orthopedic Clinic, Seoul, Republic of Korea; 4 Department of Orthopedic Surgery, College of Medicine, Hanyang University Hospital at Guri, 153, Gyeongchun-ro, Guri-si, Gyeonggi-do Republic of Korea; 5 Sports and Exercise Medicine Lab, Korea Maritime and Ocean University, Busan, Republic of Korea

**Keywords:** Knee, Meniscus, Discoid meniscus, Epidemiology, Incidence

## Abstract

**Background:**

There is a lack of evidence of the diagnosis and treatment-related epidemiological studies of symptomatic discoid meniscus. This study analyzed the national epidemiological data for discoid meniscus in South Korea.

**Methods:**

From 2011 to 2019, data related to the diagnosis and procedure codes of discoid meniscus were obtained from the Korean Health Insurance Review and Assessment Service database. All patients encoded as discoid meniscus were included. Data were extracted and further analyzed as follows: (1) the total number and the incidence (cases per 100,000) of discoid meniscus diagnosis per year, (2) sex distribution, (3) age distribution, (4) discoid meniscus ratio (total discoid meniscus coding per total meniscus injury coding), and (5) surgical procedures after discoid meniscus injury.

**Results:**

The total number of discoid meniscus diagnosed was 4576 in 2011 and increased to 6639 in 2019, representing a 45.1% increase. The incidence was 9.5 in 2011 and increased to 13.0 in 2019. Concerning sex, discoid meniscus was more common in females (55%) than in males (45%) over the study period. Regarding age, the peak age of discoid meniscus in 2011 was “under 19,” whereas in 2019, the peak age was observed in the 50s. The discoid meniscus ratio range was 2.12–2.60% from 2011 to 2019. The total number of meniscectomy increased by 20% from 2000 in 2011 to 2475 in 2014. However, the total number of meniscus repairs was 318 in 2011 and increased to 502 in 2019, indicating an increase of 58%.

**Conclusions:**

The total number and incidence of symptomatic discoid as well as the discoid meniscus ratio and the incidence of total discoid meniscus repair steadily increased from 2011 to 2019. The number of meniscus repair procedures increased more rapidly than that of meniscectomy. The current study helps understand the epidemiology of symptomatic discoid meniscus, its prevention, and cost-saving measures in South Korea.

## Introduction

The normal meniscus is crescent shaped, but the discoid meniscus is a congenital persistence of a disc-shaped meniscus [1]. A discoid meniscus has decreased collagen fibers and loss of normal collagen orientation [2], and individuals with a discoid meniscus can be asymptomatic [1]. The prevalence of discoid meniscus was reported to be 0.4–17%, 0.06–0.3%, and 20% in lateral meniscus, medial meniscus, and bilaterally, respectively [1, 2]. The incidence is higher in Asia than in Western countries [2–4].

The discoid meniscus is an intraarticular knee disorder presented in the young population and during adolescence; if not treated properly after injury, it progresses to arthritis at an early age [3]. Nonsymptomatic discoid is not considered a target for treatment, but symptomatic discoid meniscus, which is diagnosed after visiting a hospital, should be treated appropriately with suturing, if possible, to improve hoop tension, if repairable, and meniscectomy, if not repairable, to reduce mechanical symptoms such as locking and foreign body sensation [2, 5, 6].

South Korea, along with Japan, is known as one of the countries with the highest incidence of discoid meniscus worldwide [7], but there is little evidence of the exact prevalence and treatment-related epidemiological studies of symptomatic discoid meniscus. Recently, a thorough comprehension of the epidemiological patterns through big data analysis has become essential for devising effective prevention and treatment strategies [8–11]. A comprehensive epidemiological study helps prevent injuries and diseases by identifying high-risk individuals based on known risk factors and facilitates the development of targeted prevention programs for those individuals. However, to date, there is a lack of evidence of the diagnosis and treatment-related epidemiological studies of symptomatic discoid meniscus from national big data.

The aim of the present study was to analyze the national epidemiological data for discoid meniscus in South Korea from 2011 to 2019 and stratify the findings based on age, sex, and surgical procedure by analyzing the nationwide data acquired from the Korean Health Insurance Review and Assessment Service (HIRA) database. It was hypothesized that the incidence of symptomatic discoid meniscus and repair ratio increased during the study period.

## Materials and methods

This study protocol was reviewed and approved by the appropriate institutional review board (IRB) of the Inje University Seoul Paik Hospital (approval no. PAIK 2021-04-001). The IRB waived the requirements of informed consent as all data were anonymous. Thus, the study was performed without prior informed consent. The HIRA data are health insurance claims data, which is also called Korean National Health Insurance data. Thus, it is generated in the process of reimbursing claims for healthcare services under the National Health Insurance system in Korea. The HIRA data contain the medical billing data of the entire Korean population (97% health insurance and 3% medical care) [9, 12]. In South Korea, it is a legal obligation to include patient medical records in the HIRA database. Thus, surgery procedures and diagnostic codes in Korea are prospectively recorded in the HIRA data. The authors obtained and analyzed data related to procedure codes for discoid meniscus (Table 1) and surgical procedure codes (Table 2) in Korea from 2011 to 2019. All patients encoded as discoid meniscus in the HIRA database were included in this study.

**Table 1:** Diagnostic codes for discoid meniscus of the HIRA

Code	Diagnostic codes
M231	Discoid meniscus (congenital)
M2311	Discoid meniscus (congenital), medial meniscus
M2312	Discoid meniscus (congenital), lateral meniscus
M2319	Discoid meniscus (congenital), unspecified meniscus or ligament

*HIRA* Health insurance review and assessment service

**Table 2:** Diagnostic codes for surgical procedures in the HIRA

Surgical procedure	Code	Diagnostic codes
Menisectomy	N0821	Simple menisectomy in medial or lateral
	N0822	Simple menisectomy in medial and lateral
	N0826	Complex menisectomy in medial or lateral
	N0827	Complex menisectomy in medial and lateral
Meniscus repair	N0823	Simple meniscus repair in medial or lateral
	N0824	Simple meniscus repair in medial and lateral
	N0828	Complex meniscus repair in medial or lateral
	N0829	Complex meniscus repair in medial and lateral

*HIRA* Health Insurance Review and Assessment Service

In the HIRA database, the diagnostic code for discoid meniscus is classified into four categories. All patients encoded as discoid meniscus were included in this study. The discoid meniscus data were extracted and further analyzed as follows: (1) the total number of discoid meniscus diagnosed per year, (2) sex distribution of discoid meniscus in patients, (3) age distribution of discoid meniscus in patients, (4) discoid meniscus ratio (total discoid meniscus coding per total meniscus injury coding), and (5) the number of surgical procedures including meniscectomy and meniscus repair after discoid meniscus diagnosis, and repair ratio ([total number of repair/total number of meniscectomy + repair] × 100). The total number of patients per year was investigated and was standardized as the incidence of discoid meniscus per 100,000 persons per year. “Per 100,000 person-years” is simply referred to as “per 100,000 person-year” and was the only descriptive statistic used in this study. Subsequently, a more detailed analysis was performed based on sex and age. Age data were presented in decades due to the unavailability of exact ages in years. The incidence per 100,000 person-year was calculated using additional information from surveys, including basic epidemiological data from the Korean National Statistics. The incidence for each age decade was based on the total number of age decades, not the entire national population.

## Results

**Fig. 1:**
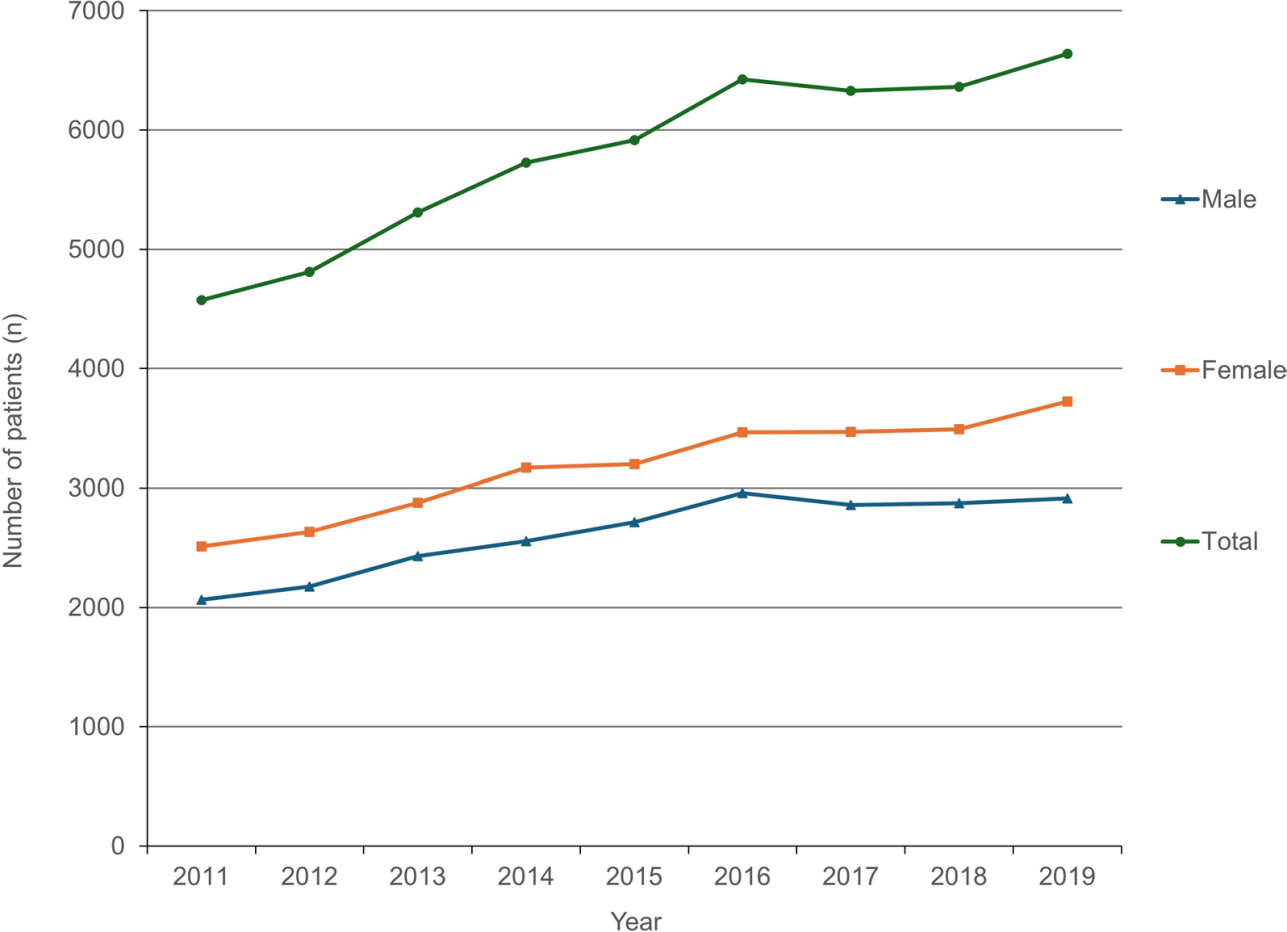
Total number of discoid meniscus patients per year stratified by sex

**Fig. 2:**
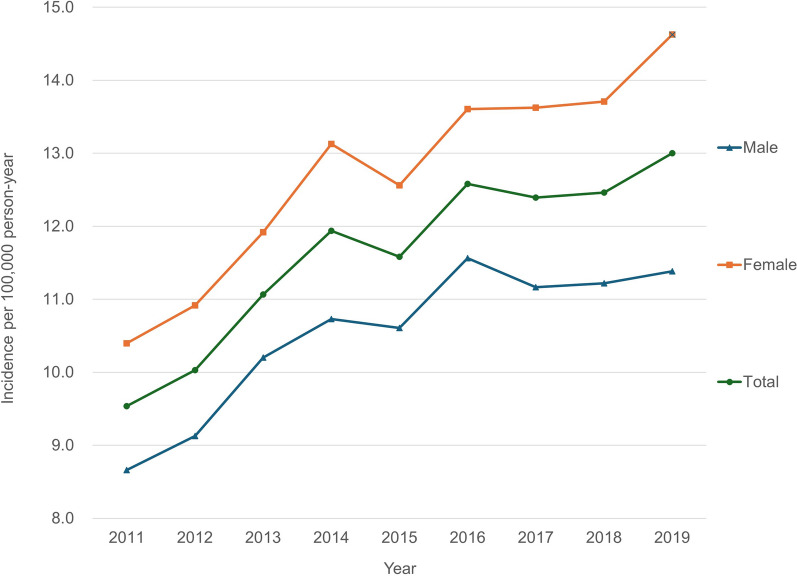
Total number of discoid meniscus patients per year stratified by age

The total number of discoid meniscus diagnoses was 4576 in 2011 and increased to 6639 in 2019 (Fig. 1), indicating that it increased by 45.1% over 9 years. The incidence per 100,000 person-year was 9.5 in 2011, which increased to 13.0 in 2019 (Fig. 2).

Regarding sex, the number of discoid menisci was greater in females (approximately 55%) than in males (approximately 45%) over the study period. Among males, the total number of patients and incidence per 100,000 person-year were 2065 and 8.7, respectively, in 2011, which increased to 2915 and 11.4, respectively, in 2019 (Figs. 1, 2). Among females, the total number of patients and the incidence per 100,000 person-year were 2511 and 10.4, respectively, in 2011, which increased to 3724, and 14.6, respectively, in 2019 (Figs. 1, 2).

**Fig. 3:**
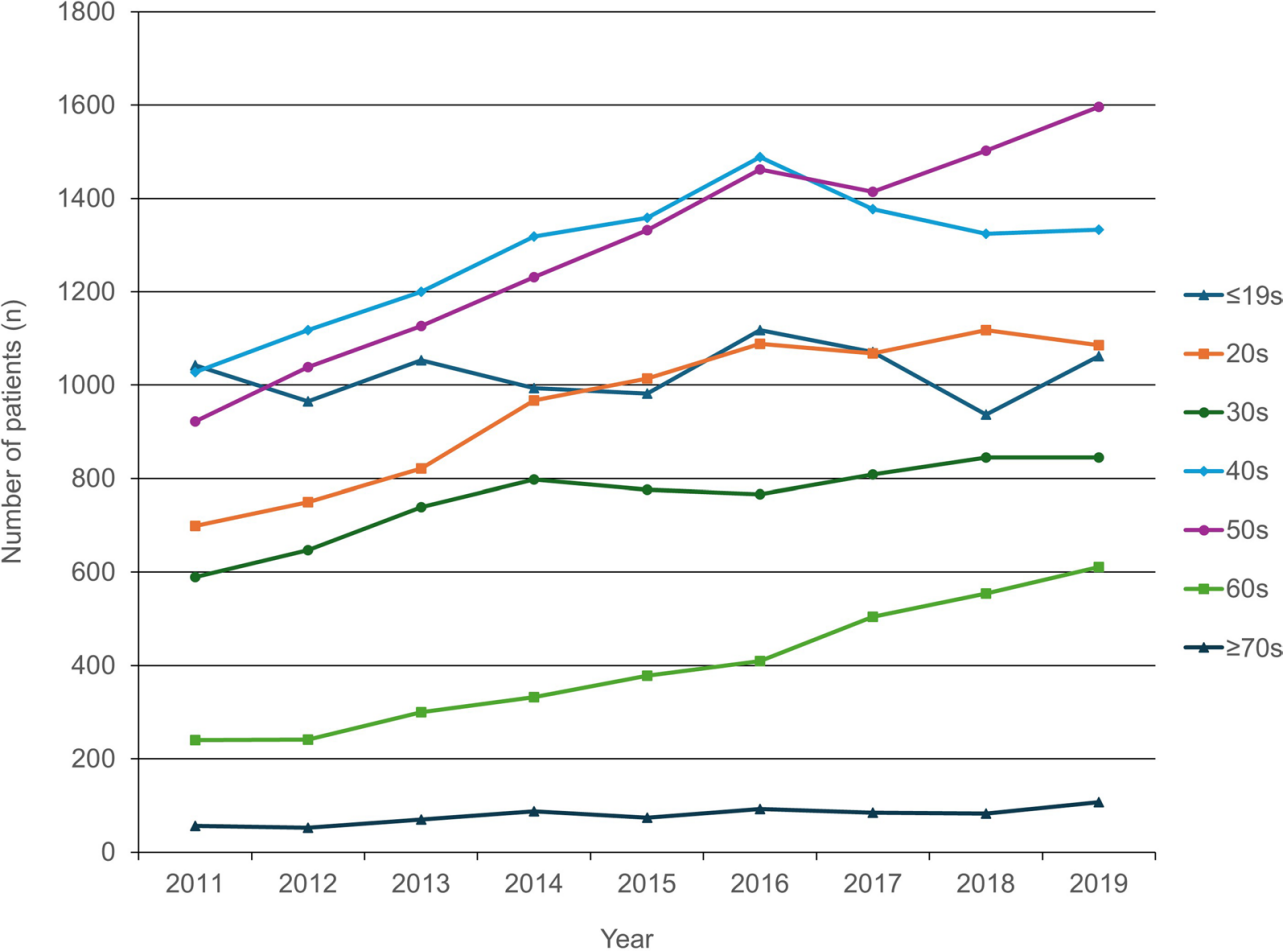
Total number of discoid meniscus patients per year stratified by age

**Fig. 4:**
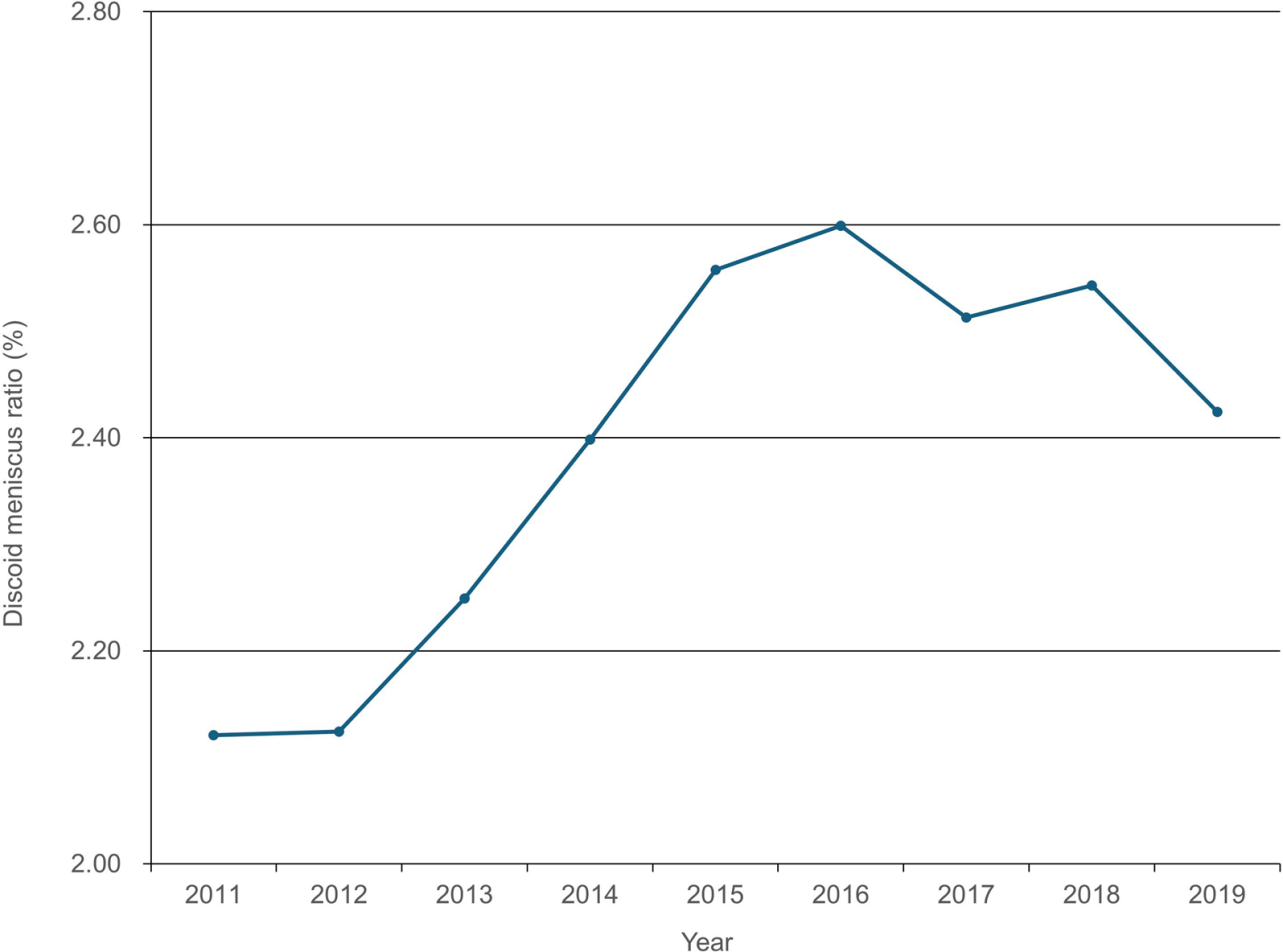
Diagnosis ratio in discoid meniscus per total number of meniscus tear

Regarding age, the peak for the total number of patients in 2011 was noted in patients who were “under 19,” whereas in 2019, the peak age was observed in their 50s, followed by 40s, 20s, and “under 19” (Fig. 3). Regarding discoid meniscus ratio, the ratio ranged from 2.12–2.60% from 2011 to 2019 (Fig. 4), increasing by 23%.

**Fig. 5:**
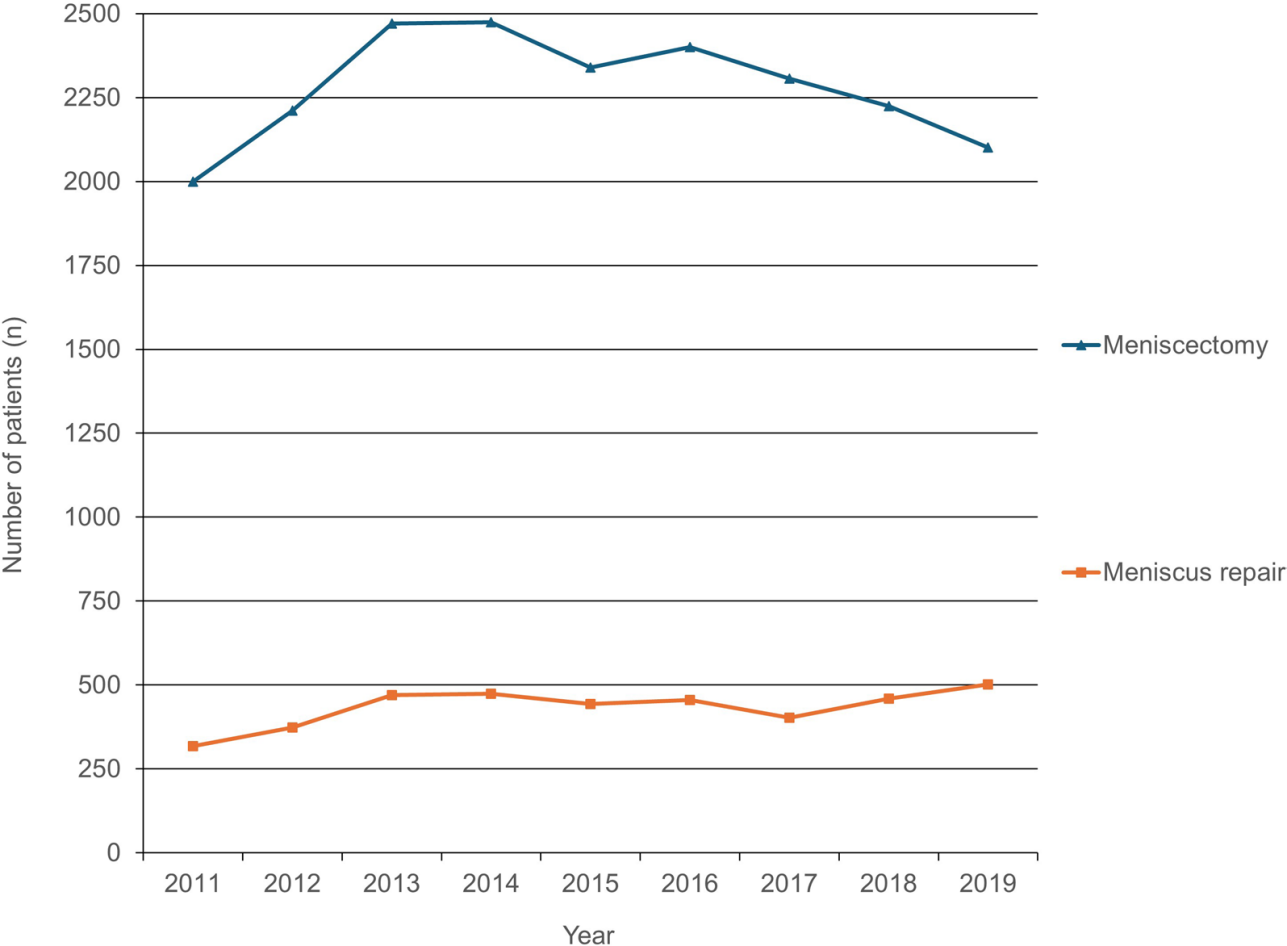
Total number of menisectomy and meniscus repair cases

Regarding the surgical procedure, the total number of meniscectomy was 2000 in 2011 and 2475 in 2014, increasing by 20%. However, the total number of meniscus repairs was 318 in 2011 and increased to 502 in 2019, an increase of 58% (Fig. 5). The repair ratio increased from 13.7% in 2011 to 19.3% in 2019. Among repair ratio data, in terms of age, teens were highest at 32.2%, followed by those in their 20s at 20.0% and those in their 30s at 15.1%.

## Discussion

The main findings of this study are as follows: (1) the total number of discoid meniscus diagnoses was 4576 in 2011, and this number increased to 6639 in 2019, indicating that it increased by 45.1% over 9 years; (2) the incidence per 100,000 person-year was 9.5 in 2011 and increased to 13.0 in 2019; (3) symptomatic discoid meniscus was diagnosed more frequently in females than in males; (4) the peaks and second peaks for the total number of patients and the incidence were observed among patients in their 50s and 40s, respectively, in 2019; (5) the number of meniscus repairs rapidly increased compared with that of meniscectomy, and the repair ratio increased from 13.7% in 2011 to 19.3% in 2019, with repairs commonly performed at relatively younger age including 10s and 20s.

Symptomatic discoid meniscus is observed commonly in patients with meniscus tears; thus, the national trends and epidemiology of symptomatic discoid meniscus are critical for future planning and national policy development. Few reports are available on the national trends and epidemiology of symptomatic discoid meniscus around the world [13–16]. Previous Korean studies have looked at discoid meniscus incidence or epidemiology [17, 18], but none of them used big data. To the best of our knowledge, this is an uncommon study to report the national trends of symptomatic discoid meniscus in Korea.

In the current study, the total number of discoid meniscus diagnoses was 4576 in 2011, and this number increased to 6639 in 2019. In the USA, the overall prevalence rate was 4.88 per 100,000 patients [13], and the overall annual incidence of symptomatic discoid lateral meniscus was 3.2 per 100,000 person-years [16]. In the Greek population, the incidence rate of discoid meniscus was 1.8% of all knee arthroscopies [15]. In this study, the total number of discoid meniscus diagnosis increased 45.1% over this 9-year period. Several reasons could be considered for this. Korea has a national insurance system that covers all Korean patients with a relatively low cost and provides easy access to orthopedic hospitals. In addition, recently, the number of diagnoses has increased because most of the Korean population has private insurance that can cover uncovered items at a high-cost national insurance, such as magnetic resonance imaging.

Concerning sex difference, the number of discoid menisci was greater in females (55%) than in males (45%) during the study period. Similar to the current study, in the USA, 56.6% of patients with discoid meniscus were females, a statistically larger proportion of the discoid meniscus population compared with the proportion of female controls in the study population [14]. In addition, the odds of discoid meniscus were 43% lower for males than females [14]. However, several studies reported a higher incidence in males [13, 16] and no statistically significant correlation between sex and lesion incidence [15]. Speculatively, the sex ratio might vary depending on the data collection period or the relationship between age and sex. However, recently in Korea, females’ participation in physical activities has been increasing [14, 19]; therefore, it could be considered that meniscus discoid injury occurs more commonly in females.

Concerning age, the peak for the total number of patients in 2019 was noted in patients who were in their 50s, followed by 40s, 20s, and “under 19.” Usually, the highest incidence of discoid meniscus was noted in adolescent male patients aged 15–18 years [16], but in this study, the peak age was patients in their 50s. Randhawa et al. reported that age is a significant predictor, with the odds of discoid meniscus decreasing by 6% for every increase of 1 year in age and with the odds of discoid meniscus decreasing by 34% for every increase of 1 year in age [14]. Grimm et al. also reported that the surgical rate decreased by approximately 6% for every 10-year increase in age [13]. In Korea, based on the fact that social activity participation is associated with physical and cognitive functions in middle-aged adults [20], their physical activity participation is increasing, and the government is also making efforts to create an environment for exercise. However, physical or sports activities are always accompanied by injuries. The reason for the increase in the number of discoid meniscus diagnoses among middle-aged people in Korea is considered because of their increasing social activities and participation.

Symptomatic discoid meniscus ratio ranged from 2.12% to 2.60% from 2011 to 2019. To the best of our knowledge, only one study confirmed the discoid meniscus ratio. Similar to our finding, In the Greek population, discoid variation was reported in 2.75% of the arthroscopies and were attributed to meniscus lesions [15]. In the USA, patients who underwent lateral meniscus repair in addition to cauterization represented 18.3% [16]. In the current study, the total number of meniscectomies steadily increased by approximately 20% over the study period, but the total number of meniscus repairs rapidly increased by about 58%. Additionally, the repair ratio increased from 13.7% in 2011 to 19.3% in 2019. According to the repair ratio data, in terms of age, teens were highest at 32.2%, followed by those in their 20s at 20.0%, and those in their 30s at 15.1%. This is an effort by surgeons to maintain meniscus hoop tension to prevent arthritic progression and preserve meniscal function. Traditionally, partial meniscectomy is generally recommended if the discoid meniscus is associated with mechanical symptoms, such as pain, locking, swelling, giving way, or causing an inability to participate in sports [5]. Owing to the success rate of arthroscopic meniscal repair techniques for partial central meniscectomy in the vascular zone [21], the operation frequency of Korean surgeons is increasing in middle-aged patients with meniscus root tears, and interest in root repair has been increasing [22]. Thus, the total number of meniscus repairs seems to have increased. Especially, the high rate of repair at younger ages suggests that more effort is being made to restore meniscal hoop tension at younger ages. The current study did not include statistical analysis and statistical significance from a small sample size, because it was based on patients with total discoid meniscus. In any experiment or observation involving drawing a sample from a population, observed results from a small sample may be generalized to the entire population through statistical analysis. However, there was no need to conduct such a statistical analysis in this study.

The importance of this study is that it is a valuable report to investigate the national trends of discoid meniscus based on a national database in Korea. This national database is one of the most accurate national databases in the world as it covers the entire Korean population, includes all medical records, and is controlled by the Korean government.

However, several limitations exist in this study. First, there is the possibility of coding errors in a large database. Second, since our data are an analysis of discoid meniscus presenting to the hospital with knee pain, it may include asymptomatic discoid meniscus as well as symptomatic discoid meniscus. The results may be different than if only symptomatic discoid meniscus or asymptomatic discoid meniscus were examined. Third, discoid meniscus in our analysis did not include subjects injured in traffic accidents, industrial accidents, or those who made claims for automobile liability insurance or industrial injury insurance; however, there are potentially only a few of these patients in comparison with the data recorded in the HIRA database. Fourth, the meniscus surgery numbers we analyzed included only meniscus surgery coding; thus, other concomitant knee surgeries could not be investigated. Fifth, patient diagnosed with bilateral discoid meniscus and those who underwent bilateral meniscus procedures could not be identified, because this study was based on a large-scale database. Thus, this study presented the number of patients and not the number of cases. Sixth, there is a possibility that many cases of discoid meniscus were coded as meniscus tears as some insurance programs only reimbursed traumatic tears. Therefore, the numbers of discoid meniscus cases (especially those with tears) are likely to have been underreported in the data. Additionally, the coding of the diagnosis did not always signify the symptomatic state of the discoid meniscus. Seventh, most discoid meniscus cases are congenital in nature; thus, the incidence rates should be similar regardless of age and frequency. However, in this study, the incidence rates of discoid meniscus differed based on age and frequency. This is assumed to be because of differences in hospital accessibility, hospital presence, and diagnostic performance. This phenomenon is a common limitation in big data analysis studies. Eighth, in this study, as coded patients were judged to have symptoms, they were defined as patients with symptomatic discoid meniscus. This may be a limitation of this study because some of the coded patients may have had no symptoms and were diagnosed coincidentally. However, the numbers of these patients are limited and unlikely to significantly affect the results. Discoid lateral meniscus is more commonly noted and clinically important. However, this study did not differentiate discoid lateral meniscus from discoid medial meniscus. Further research should focus on the discoid lateral meniscus.

## Conclusions

The total number and incidence of symptomatic discoid and the discoid meniscus ratio steadily increased from 2011 to 2019. The number of meniscus repair procedures increased more rapidly than that of meniscectomy. The current study helps understand the epidemiology of symptomatic discoid meniscus, its prevention, and cost-saving measures in South Korea.
